# Supplementary material for: Extracellular Vesicles from Cerebrospinal Fluid of Leptomeningeal Metastasis Patients Deliver MiR-21 and Induce Methotrexate Resistance in Lung Cancer Cells
Source: Int J Mol Sci. 2024 Mar 8;25(6):3124. doi: 10.3390/ijms25063124 (PMC10970033; doi:10.3390/ijms25063124)

Supporting Information for

Extracellular Vesicles from Cerebrospinal Fluid of Leptomeningeal Metastasis Patients Deliver MiR-21 and Induce Methotrexate Resistance in Lung Cancer Cells

Ji Hye Im [1], Kyue-Yim Lee [1], Yoona Seo [2,3], Jiho Rhim [2,3], Yun-Sik Dho [4], Byong Chul Yoo [3], Jong Bae Park [3], Sang Hoon Shin [4], Heon Yoo [3,4], Jong Heon Kim [2,3,*] and Ho-Shin Gwak [1,4,*]

[1]Department of Cancer Control, National Cancer Center Graduate School of Cancer Science and Policy, Goyang 10408, Republic of Korea. [2]Cancer Molecular Biology Branch, Research Institute, National Cancer Center, Goyang 10408, Republic of Korea. [3]Department of Cancer Biomedical Science, National Cancer Center Graduate School of Cancer Science and Policy, Goyang 10408, Republic of Korea. [4]Neuro-Oncology Clinic, National Cancer Center, Goyang 10408, Republic of Korea.

[*]Correspondence: jhkim@ncc.re.kr (J.H.K.); nsghs@ncc.re.kr (H.-S.G.)

This PDF file includes:

Supplementary Materials and Methods
Supplementary Results
Supplementary Figures S1 to S5
Supplementary Tables S1 to S2
Supplementary References
Uncropped Images: Figure Immunoblots

Supplementary Materials and Methods

Cell culture

Various human lung cancer cell lines were obtained from Hyonchol Jang (A549, HCC827, and HOP-62; National Cancer Center, Korea) and Soo-Youl Kim [NCI-H226 (H226), NCI-H460 (H460), and NCI-H1299 (H1299); National Cancer Center, Korea]. Lenti-X 293T cells were purchased from Takara Bio Inc. (Shiga, Japan). Cell lines were cultured in Dulbecco's modified Eagle medium (DMEM; Cytiva, Marlborough, MA, USA) and RPMI 1640 (Cytiva) supplemented with 10% fetal bovine serum (Cytiva), 1% penicillin/streptomycin (Welgene, Gyeongsan, Korea), and 10 mg/ml ciprofloxacin (Santa Cruz Biotech, Santa Cruz, CA, USA).

Real-time reverse transcription-PCR of cellular miR-21

Total RNA was isolated from samples using TRIzol™ LS reagent (Thermo Fisher Scientific, Waltham, MA, USA) according to the manufacturer's protocol. The samples were added to 0.75 ml TRIzol™ LS reagent and 0.2 ml chloroform (Sigma-Aldrich, St. Louis, MO, USA) and centrifuged for 30 min at 12,000× g at 4 °C. The aqueous phase was transferred to a new tube, supplemented with 0.5 ml isopropanol, and incubated overnight at – 20 °C. The sample was then centrifuged for 30 min at 12,000 × g at 4 °C. The RNA pellet was then washed with 75% ethanol, dried, and resuspended in 20 μl RNase-free water. One microgram of total RNA was used to synthesize cDNA using the TaqMan® microRNA Reverse Transcription kit (cat. 4366597, Applied Biosystems, Waltham, MA, USA) and TaqMan® MicroRNA Assay RT primer (miR-21-5p ID: 000397, U6 snRNA ID: 001973, Applied Biosystems). The cDNA was mixed with TaqMan® MicroRNA Assay primer and TaqMan® Universal Master Mix (cat. 4324018, Applied Biosystems), and real-time PCR was performed using the QuantStudio™ 7 Flex Real-Time PCR System (Applied Biosystems). The relative miR-21 expression was normalized to U6 expression.

Digital droplet PCR of EV-derived miR-21

Extracellular miR-21 expression in CSF was determined using a droplet digital PCR (ddPCR) system as previously described [1]. Total RNA was isolated using a microRNA Purification Kit (Norgen Biotek, ON, Canada) or mirVana PARIS (Ambion, Thermo Fisher Scientific) according to the manufacturers' instructions. About 2 ng purified total RNA was used to synthesize cDNA with a TaqMan Advanced miRNA cDNA Synthesis Kit (A28007, Applied Biosystems) according to the manufacturer's instructions. Four microliters of cDNA product were mixed with ddPCR Supermix for Probes (Bio-Rad Laboratories, Hercules, CA, USA) and TaqMan miRNA Assay probes (Applied Biosystems; hsa-miR-21-5p). Each 20 μl reaction mixture was mixed with 70 μl droplet-generation oil and partitioned into 20,000 nanoliter-sized droplets by a QX299 droplet generator (Bio-Rad Laboratories). A final 40 μl droplet mixture was used for PCR as follows: 95 °C for 5 min (enzyme activation), 40 cycles of 95 °C for 30 s (denaturation) and 55°C for 1 min (annealing), and a final step of 98 °C for 10 min (enzyme inactivation). The amplified PCR product was analyzed for gene concentration (copies/nl) using a QC200 Droplet Reader (Bio-Rad Laboratories) and built-in QuantaSoft v.1.7.4.0917 software (Bio-Rad Laboratories). Finally, the gene concentration was corrected for the input CSF volume.

EV marker quantification

EV-specific markers were determined by flow cytometry with a MACSPlex Exosome Kit (Miltenyi Biotec, Gladbach, Germany) per the manufacturer's protocol. Briefly, CSF or culture medium and 15 μl barcoded exosome capture beads were incubated overnight on an orbital shaker and washed with 0.5 ml MACSPlex buffer (MPB) three times. The samples were then supplemented with a 15 μl detection reagent mixture (APC-conjugated anti-CD9, anti-CD63, and anti-CD81 antibodies) and incubated for 2 h. After washing with 0.5 ml MPB three times, the beads were resuspended in 0.2 ml MPB buffer and analyzed with a BD LSRfortessa™ (BD Biosciences, San Jose, CA, USA). Exosome setup beads were used to set up the instruments, and flow cytometry data were analyzed using FlowJo software (ver. 10). Single beads were gated, and median fluorescence intensity (MFI) values of barcoded bead populations were used to determine the expression of exosome markers. EV-specific markers and size were determined by ExoView kit assay (NanoView Bioscience, Boston, MA, USA) and an ExoView R100 system (NanoView Bioscience) per the manufacturer's protocol. An anti-CD9, anti-CD63, and anti-CD81 pre-coated chip was pre-scanned before the validation and incubated with experimental samples overnight. The chip was then washed with PBST three times and incubated with 250 μl anti-CD9 (CF 488A), anti-CD81 (CF 555), and anti-CD63 (CF 647) mixture. The chip was then washed three times with deionized water, air-dried, and scanned by an ExoView R100. Scanned data were analyzed using ExoView analyzer software (ver. 3.1.4, NanoView Bioscience).

PKH26 labeling and cellular uptake of EV

EV were labeled with the PKH26 Red Fluorescent Kit (Sigma-Aldrich) to verify their uptake into recipient cells. After EV isolation by ultracentrifugation, EV were resuspended in 0.1 ml Diluent C solution, and 1 μl PKH26 solution was added to 0.1 ml Diluent C to prepare a 4×10^{-6} M dye solution. The EV suspension was mixed with the dye solution for 5 min, after which 0.2 ml of 10% bovine serum albumin was added to quench the reaction. The labeled EV were centrifuged at 116,000× g for 2 h and incubated with H460 cells overnight. The cells were then fixed with 4% paraformaldehyde, and the nuclei were stained with DAPI. EV uptake was observed under an LSM 780 Confocal microscope (Zeiss, Oberkochen, Germany).

Supplementary Results

Isolation and characterization of EVs in cell culture media

Given the evidence that EV carry miRNAs and contribute to cancer development and progression, we hypothesized that EV containing miR-21 contribute to MTX resistance in NSCLC cells [1-3]. To test this, we characterized EV isolated from A549 cell culture media. Because FBS is known to contain a large amount of EV, A549 cells were cultured in serum-free medium for 2 to 3 days and the supernatant was harvested and EV pellets were precipitated by ultracentrifugation. The particle size distribution of the EV measured by NTA and the mean particle size was 133.8 ± 1.6 nm (Supplementary Figure 3A). To verify EV surface markers in the pellets, we measured CD9, CD63, and CD81 using both a MACSPlex kit, which uses beads tagged with anti-CD9, -CD63, or -CD81 to capture EV in liquid samples, and the ExoView platform, which uses the same antibodies pre-coated to a sensor chip. The results confirmed the expression of EV markers in the A549 EV pellets (Supplementary Figures 3B and 3C).

Supplementary Figures

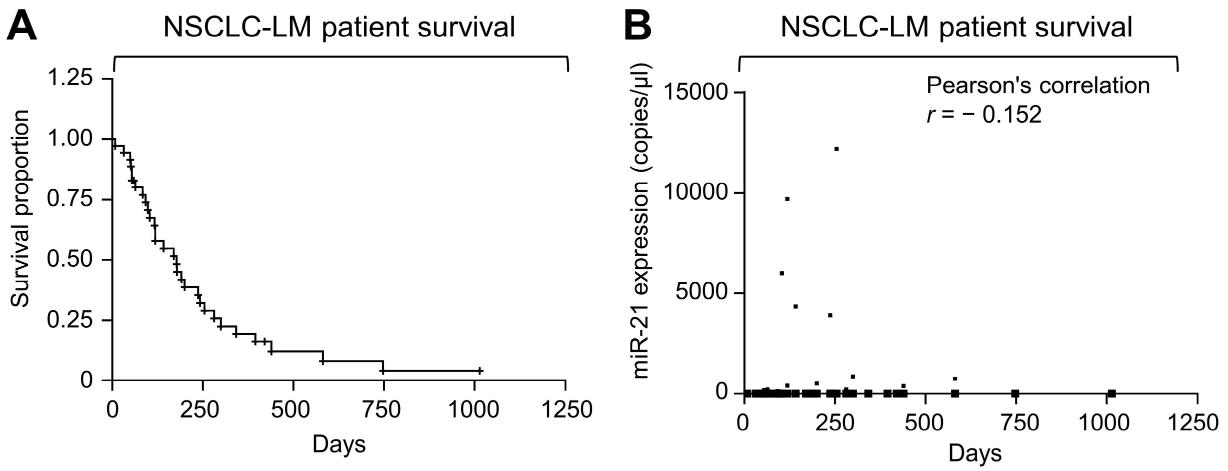

Supplementary Figure S1. Survival and CSF extracellular miR-21 levels of patients with leptomeningeal metastasis. (**A**) Kaplan-Maier plot for 36 patients with NSCLC-LM who received ventriculolumbar perfusion methotrexate chemotherapy. Survival days were estimated from LM diagnosis to the last follow-up date. Median overall survival was 237 days (range, 191−395 days). (**B**) CSF miR-21 expression levels of individual patients were plotted against overall survival. Correlation analysis was performed using Prism (ver. 3.0).

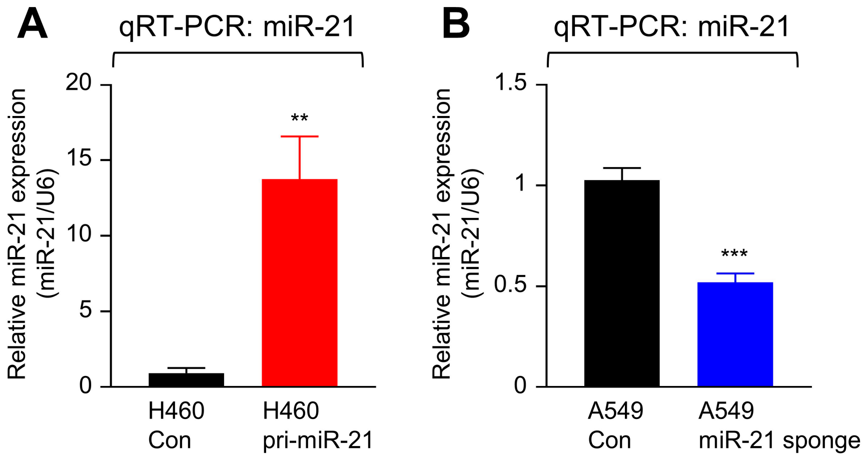

Supplementary Figure S2. Quantification of miR-21 in pri-miR-21 or miR-21 sponge-expressing cell lines by qRT-PCR. (**A**) Transduction of pri-miR-21 minigene-expressing lentiviral system in H460 cell caused a 14.8-fold increase in miR-21-5p expression compared with that in control H460 cell. (**B**) Transduction of miR-21 sponge-expressing lentiviral system reduced miR-21-5p expression around 48% in A549 cell. Error bars in the graph represent ± standard deviation and statistical significance was verified by two-tailed Student's *t*-test ($n = 4$). $^{**}p < 0.01$, $^{***}p < 0.001$.

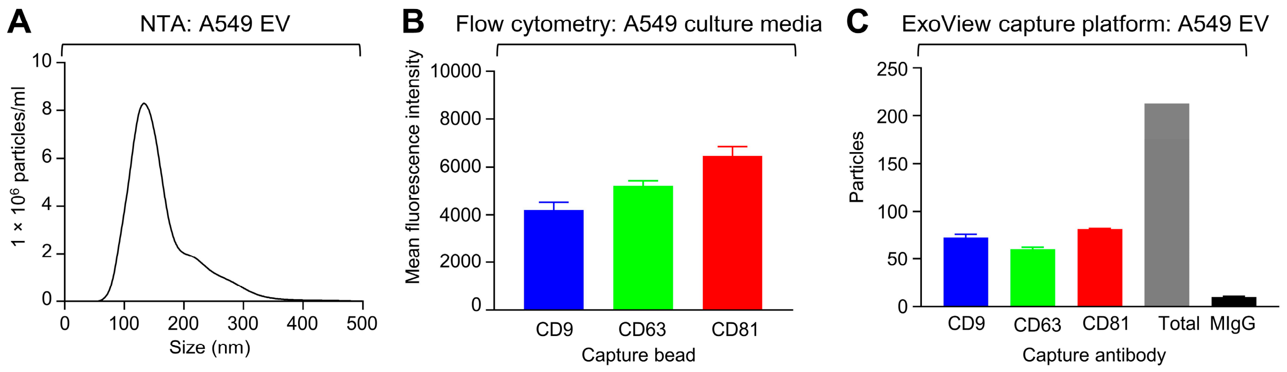

Supplementary Figure S3. Characterization of EVs isolated from NSCLC cell culture media. (**A**) The size distribution and concentration of EVs isolated from cell culture media were measured by Nanoparticle Tracking Analysis. (**B**) EV surface markers were confirmed by a MACSPlex kit with CD9, -CD63, or -CD81 capturing beads. Error bars in the graph represent ± standard deviation. (**C**) EV surface markers were confirmed by the ExoView platform, in which these antibodies were pre-coated to a sensor chip. Error bars in the graph represent ± standard deviation.

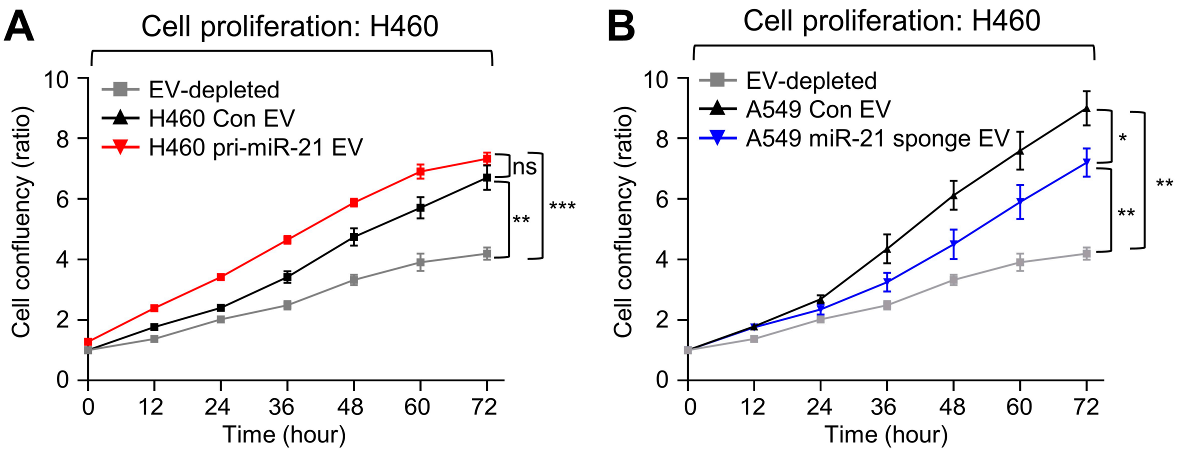

Supplementary Figure S4. Delivery of EVs derived from pri-miR-21- or miR-21 sponge-expressing NSCLC cells can modulate cell proliferation in H460 cells. (**A**) H460 cells were treated with H460 EV or pri-miR-21 H460 EV. (**B**) H460 cells were treated with A549 EV or miR-21 sponge A549 EV. Relative confluence to 0 h was calculated at 72 h using IncuCyte software. Error bars in the graph represent ± standard deviation and statistical significance was determined by two-tailed Student's *t*-test (n = 3).

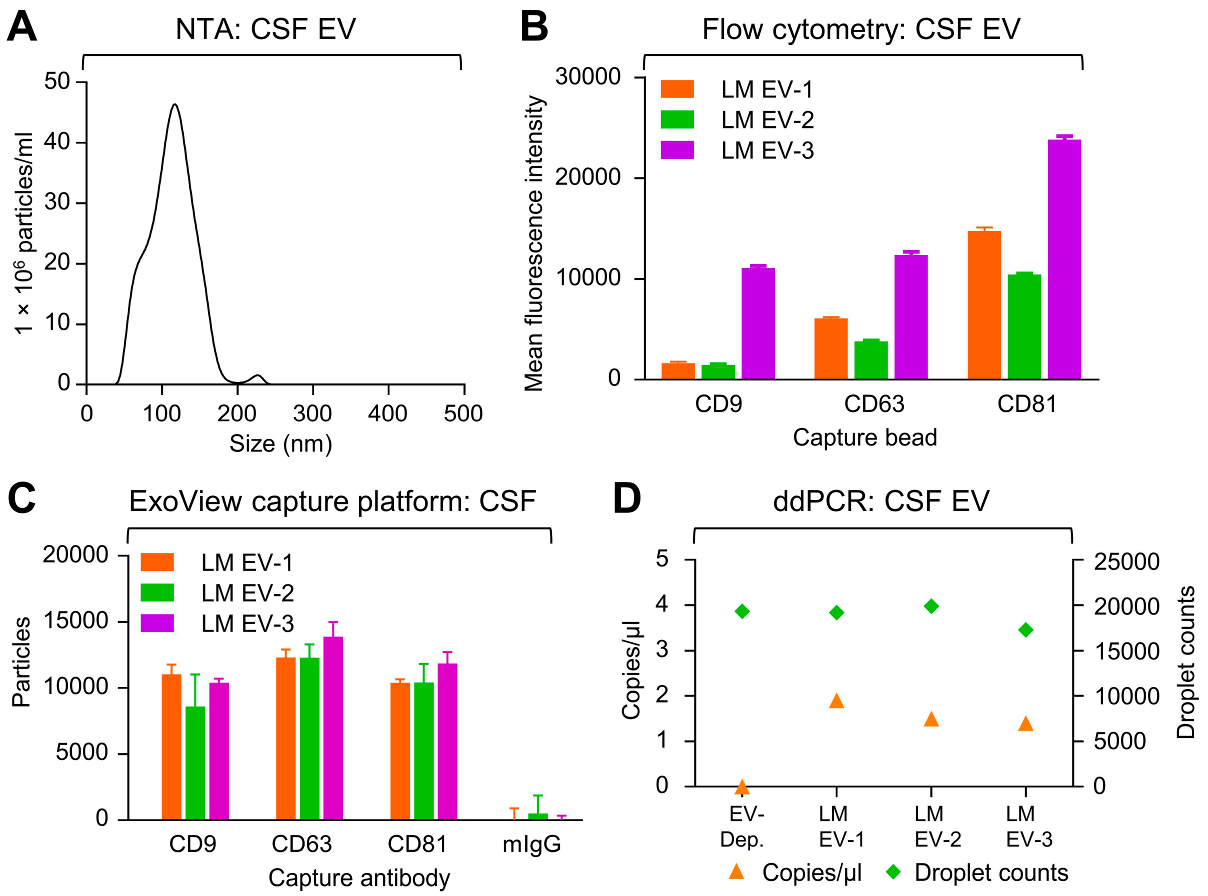

Supplementary Figure S5. Characterization of CSF EVs isolated from LM patients. (**A**) The size distribution and concentration of CSF EV isolated from patients with NSCLC-LM were measured by Nanoparticle Tracking Analysis. (**B**) EV surface markers were confirmed by a MACSPlex kit with CD9, CD63, or CD81 capturing beads. (**C**) EV surface markers were confirmed by the ExoView platform, in which these antibodies were pre-coated to a sensor chip. (**D**) Quantification of CSF EV miR-21 level by ddPCR. Error bars in the graph represent ± standard deviation.

Supplementary Tables

Supplementary Table S1. Demographic profile of CSF samples for small RNA seq (*n* = 10)

Sample id.	Gender	Age	Disease	Sample site
LM1	Female	67	NSCLC	Intraventricular
LM2	Female	67	NSCLC	Lumbar
LM3	Female	63	NSCLC	Lumbar
LM4	Male	44	NSCLC	Lumbar
LM5	Female	54	NSCLC	Lumbar
LM6	Male	54	NSCLC	Lumbar
LM7	Male	69	NSCLC	Lumbar
HC1	Female	61	Unruptured aneurysm	Cisternal
HC2	Female	60	Unruptured aneurysm	Cisternal
HC3	Male	59	Unruptured aneurysm	Intraventricular

LM, leptomeningeal metastasis; HC, healthy control; NSCLC, non-small cell lung cancer

Supplementary Table S2. Clinical characteristics of LM patients in the study (*n* = 36)

Patient id.	Gender	Age	Primary Cancer	Sample site	Overall survival (days)	miR-21 expression level (copies/μl)
LM1	Female	54	NSCLC, ADC	Lumbar	255	12198
LM2	Male	56	NSCLC, ADC	Lumbar	119	9700
LM3	Female	63	NSCLC, ADC	Ventricle	104	6000
LM4	Female	46	NSCLC, ADC	Lumbar	142	4350
LM5	Female	43	NSCLC, ADC	Lumbar	237	3910
LM6	Female	67	NSCLC, ADC	Lumbar	300	870
LM7	Female	54	NSCLC, ADC	Lumbar	581	749
LM8	Male	62	NSCLC, ADC	Lumbar	200	515
LM9	Male	69	NSCLC, ADC	Lumbar	119	399
LM10	Female	53	NSCLC, ADC	Lumbar	439	382
LM11	Male	71	NSCLC, ADC	Lumbar	281	216
LM12	Male	54	NSCLC, LCNE	Ventricle	64	211
LM13	Female	71	NSCLC, ADC	Ventricle	54	189
LM14	Male	62	NSCLC, ADC	Lumbar	92	132.7
LM15	Male	61	NSCLC, ADC	Lumbar	191	89.9
LM16	Female	64	NSCLC, ADC	Lumbar	35	45.1
LM17	Female	52	NSCLC, ADC	Lumbar	54	42.5
LM18	Male	37	NSCLC, ADC	Lumbar	95	26.7
LM19	Male	78	NSCLC, LCNE	Lumbar	32	25.6
LM20	Male	98	NSCLC, ADC	Lumbar	98	19.9
LM21	Male	63	NSCLC, ADC	Lumbar	8	19.1
LM22	Male	62	NSCLC, ADC	Lumbar	747	19
LM23	Female	53	NSCLC, ADC	Lumbar	117	8.2
LM24	Male	65	NSCLC, ADC	Ventricle	52	8
LM25	Female	69	NSCLC, ADC	Ventricle	84	7.9
LM26	Male	53	NSCLC, ADC	Lumbar	170	7
LM27	Male	68	NSCLC, ADC	Lumbar	179	5.7
LM28	Female	61	NSCLC, ADC	Ventricle	342	5.6
LM29	Female	48	NSCLC, ADC	Ventricle	60	5.2
LM30	Male	61	NSCLC, ADC	Lumbar	242	4.5
LM31	Male	62	NSCLC, ADC	Lumbar	68	3.5
LM32	Male	68	NSCLC, ADC	Ventricle	421	3.1
LM33	Female	63	NSCLC, ADC	Lumbar	50	1.35
LM34	Male	44	NSCLC, ADC	Lumbar	395	0.07
LM35	Male	65	NSCLC, ADC	Ventricle	178	0
LM36	Female	64	NSCLC, ADC	Lumbar	1014	0

ADC, adenocarcinoma; LCNE, large cell neuroendocrine carcinoma; LM, leptomeningeal metastasis; NSCLC, non-small cell lung cancer; VLP, ventriculolumbar perfusion

Supplementary References

1. Lee, K. Y.; Im, J. H.; Lin, W.; Gwak, H. S.; Kim, J. H.; Yoo, B. C.; Kim, T. H.; Park, J. B.; Park, H. J.; Kim, H. J.; Kwon, J. W.; Shin, S. H.; Yoo, H.; Lee, C., Nanoparticles in 472 Human Cerebrospinal Fluid: Changes in Extracellular Vesicle Concentration and miR-21 Expression as a Biomarker for Leptomeningeal Metastasis. *Cancers* **2020,** 12, 2745.
2. Lee, K. Y.; Seo, Y.; Im, J. H.; Rhim, J.; Baek, W.; Kim, S.; Kwon, J. W.; Yoo, B. C.; Shin, S. H.; Yoo, H.; Park, J. B.; Gwak, H. S.; Kim, J. H., Molecular Signature of Extracellular Vesicular Small Non-Coding RNAs Derived from Cerebrospinal Fluid of Leptomeningeal Metastasis Patients: Functional Implication of miR-21 and Other Small RNAs in Cancer Malignancy. *Cancers* **2021,** 13, 209.
3. Yu, X.; Odenthal, M.; Fries, J. W., Exosomes as miRNA Carriers: Formation-Function-Future. *Int. J. Mol. Sci.* **2016,** 17, 2028.

Uncropped Images: Figure Immunoblots

Figure 3

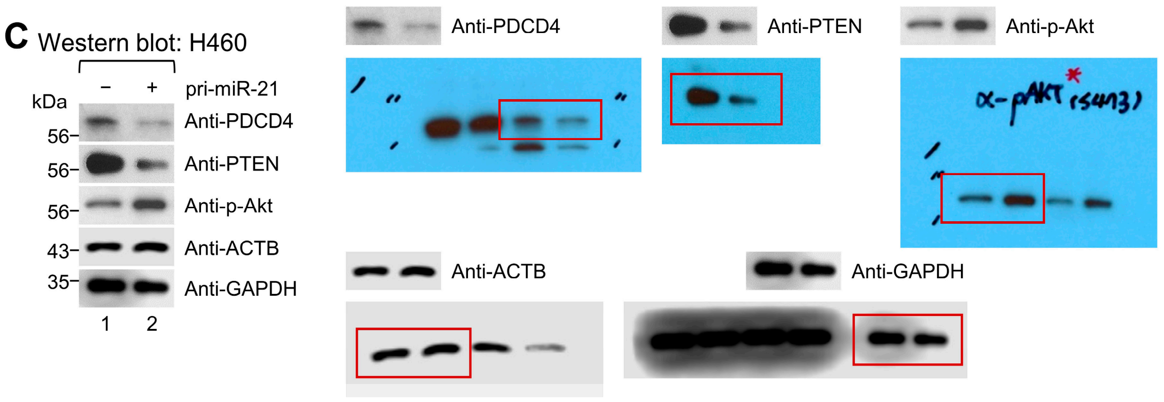

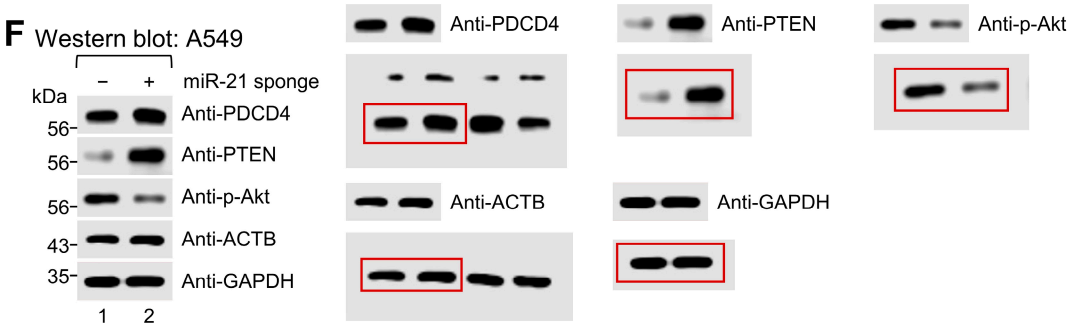

Figure 6

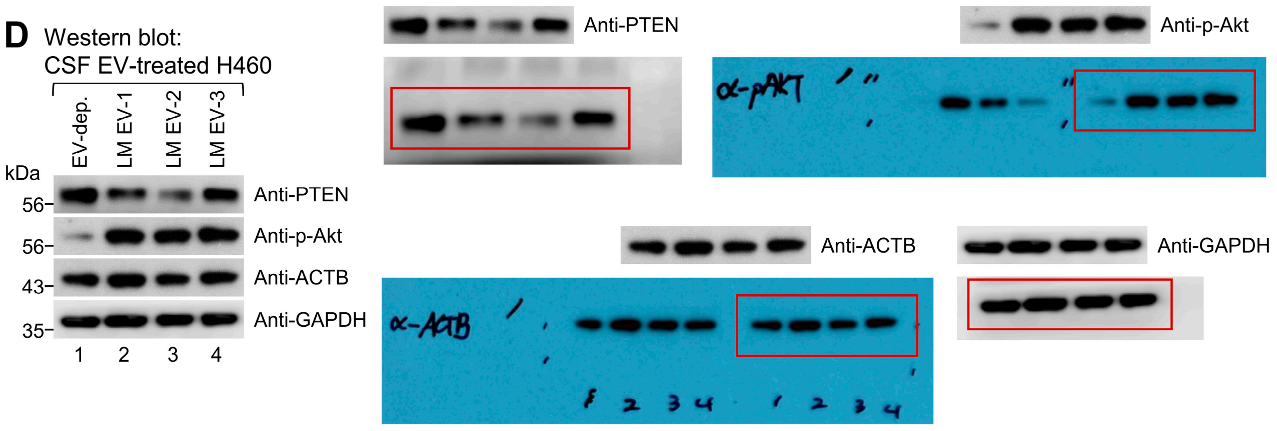

Supplement: Supplementary file 1 [file ijms-25-03124-s001.zip › ijms-2869462-supplementary.pdf]
